# Visual and lesion descriptor fusion using cross-attention for skin disease diagnosis

**DOI:** 10.3389/fonc.2026.1885137

**Published:** 2026-08-21

**Authors:** Maira Afzal, Jamal Hussain Shah, Rabia Saleem, Muhammad Usman Younas, Ali Tahir, Mutaz Elradi S. Saeed

**Affiliations:** 1 Department of Computer Science, COMSATS University Islamabad, Wah Cantt, Pakistan; 2 Department of Information Technology, Government College University Faisalabad, Faisalabad, Pakistan; 3 Department of Computer Science, College of Engineering and Computer Science, Jazan University, Jazan, Saudi Arabia; 4 Department of Computer Science, Nile Valley University, Khartoum, Sudan

**Keywords:** BERT, explainable artificial intelligence (XAI), medical imaging, multimodal, skin disease, visual transformer (ViT)

## Abstract

**Introduction:**

Accurate and early diagnosis of skin disease remains a significant challenge in clinical dermatology due to variation in skin tones and visual similarity among disease lesion types. Dermatologists rely on both clinical expertise and advanced diagnostic tools to achieve reliable diagnosis. To assist in the early diagnosis, many researchers have proposed traditional computer vision methods; however, these approaches remain limited due to the wide range of conditions and the overlapping of early-stage symptoms. Recognizing these limitations, deep learning models have been explored to improve performance, but they still face challenges in effective feature extraction and have weak generalization due to limited datasets.

**Methods:**

To address this, a cross-attention-based framework that integrates visual with structured lesion descriptor derived from dermoscopic attributes is proposed to enhance early-stage skin disease diagnosis. Visual features are extracted using a fine-tuned transformer, while descriptor features are encoded using BERT-based language models. Furthermore, the ISIC 2018 dataset is enriched with structured lesion descriptors automatically generated from dermoscopic images using the ABCD rule. The symptom descriptors provide semantic representations for multimodal framework instead of an independent clinical modality.

**Results:**

The experimental results demonstrate that the proposed framework outperforms well compared to unimodal baselines, where CNN-based methods achieved 85% to 93% accuracy, while transformer-based pre-trained models reach 94.77%. In contrast, the proposed model achieves a peak accuracy of 96.87% accuracy, which highlights its effectiveness for skin disease classification.

**Discussion:**

Unlike the traditional image-only system, the proposed model analyzed both visual features with their descriptive structured lesion descriptors that enhance interpretability and provide clinically meaningful diagnostic support.

## Introduction

Skin diseases represent one of the significant challenges in the healthcare sector that affect millions of people worldwide (1). These diseases include a wide range of dermatological conditions that differ in their symptoms, severity, and causes. Some commonly observed diseases in clinical practices include melanoma, eczema, benign, carcinoma, dermatomyositis, and keratosis (2). Although these diseases differ in their underlying causes, they have similar structure like uneven texture, lesion size, asymmetric borders, and irregular pigmentation (3). These visual similarities among skin diseases make it difficult to distinguish one skin disorder from another during clinical evaluation. As a result, in some cases, dermatologists’ prior knowledge and visual assessment alone are not always sufficient when the lesions are in their early stages and show only subtle features (4). Therefore, they frequently require advanced diagnostic tools and laboratory analysis to identify the disease type and determine its progression stage (5). Despite these challenges, precise and reliable diagnosis of skin lesion is important because it can reduce a patient’s disease progression and improve patients’ health. Some lesions may become malignant if left undetected at an early stage (6). These challenges motivate researchers to develop advance computer-aided decision support systems to assist dermatologists in their clinical practices.

Many researchers have proposed traditional computer vision methods to assist dermatologists in the initial stage of diagnosis. However, these approaches remain limited due to the wide range of conditions and the overlapping of early-stage symptoms (7). Even with the effectiveness of these techniques, precise classification and detection of skin lesions are still a significant challenge (8). Recognizing this limited accuracy, deep learning models have been explored to improve performance, but they still face challenges in effective feature extraction and have weak generalization due to limited datasets. Therefore, researchers have focused on advanced computer vision and deep learning diagnostic methods that provide a cost-effective alternative, but the analysis of skin lesion images remain a challenge because of their variation in appearance and characteristics (9). Unlike general object classification techniques, skin lesion images have high inter-class similarity and often lack uniform spatial patterns. This makes distinguishing red, patchy eczema regions and accurate feature extraction a significant challenge (10). Moreover, factors such as low contrast between the affected area and surrounding skin, irregular or blurred edges, and discoloration within wounds further complicate the recognition of acute diseases. This inability contribute to delayed diagnosis, which negatively influences a patient’s psychological and social wellbeing (11). Skin lesion identification is comparatively neglected at early stages, which can lead toward deadly cancer over time. To overcome this gap, artificial intelligence (AI)-based diagnostic systems that analyze large-scale clinical images and assist dermatologists in decision making have been developed. However, these recent AI-driven systems rely on visual signals and overlook contextual clinical descriptors such as lesion size, color, texture, and its associated symptoms. These factors restrict model interpretability and generalization that are particularly important for reliable early-stage detection. To address this, a multimodal AI-based diagnostic framework that integrates visual features (input images) with textual structured lesion descriptors and suggests a possible diagnosis based on learned patterns is proposed. Unlike the traditional image-only model, the proposed model analyzed both visual features with their descriptive metadata that enhance interpretability and provide a clinically meaningful diagnostic support. It is aimed to achieve more accurate and clinical interpretation as well as reduce bias through the combination of feature selection with semantic analysis to improve diagnoses. Additionally, this system not only assists early disease detection and patient screening but also provides dermatologists a second opinion, which enhances their diagnostic confidence and serves as a diagnostic support tool. The key contributions of this research are as follows:

- To develop a dermatology-guided architecture by extracting structured lesion descriptors according to ABCD criteria on dermoscopic images that encompass clinically sensitive features like asymmetry, border irregularity, color variation, lesion size, erythema, and texture.
- To design and implement a cross-modal attention mechanism that integrates structured lesion descriptors with deep visual analysis to align complementary representations for accurate and robust skin disease diagnosis.
- To employ a fine-tuned Vision Transformer for capturing high-level visual features from dermoscopic images and the BERT language model for extracting structured semantic embeddings, enabling effective multimodal feature representation to enhance classification accuracy and generalization compared to traditional CNN models.
- To construct a descriptor-enhanced dataset by integrating images from publicly available ISIC 2018 datasets with corresponding structured lesion attributes that enable supervised multimodal learning with domain-informed features.

Overall, the proposed research is an attempt to overcome the drawbacks of visual diagnostic system alone and develop a descriptor-guided, domain-informed learning framework by combining structured lesion descriptors and a visual representation of deep models. This proposed approach does not rely only on raw image attributes but incorporates clinically meaningful attributes generated based on dermatological criteria, which allows presenting features more correctly and consistently. Unlike traditional image-only techniques, the proposed model integrate structured lesion descriptors with visual information and provide a semantic representation instead of independent clinical modality. The framework employs transformer-based models to learn both global visual feature and structured semantic content. Moreover, cross-attention is applied to efficiently align these two feature representations. It has been demonstrated by experimental results that this integration improves diagnostic performance than visual-only models and offers a more clinical interpretation of skin lesions.

The remaining section of this paper is organized in the following order: Section 2 reviews the state-of-the-art-related skin disease articles from different domains. Section 3 describes the creation of the proposed lesion-enhanced structural descriptor. Section 4 presents step by step the proposed methodology in detail. Section 5 describes the detailed experimental results and evaluation. Section 6 concludes with the findings, limitations, and further research directions.

## Related work

Recent advances in deep learning and medical imaging significantly improve the existing skin cancer detection techniques and analysis of lesions. Convolutional neural networks (CNNs) remain the most widely used architecture for skin lesion classification and segmentation because they have the capability to extract useful features. Utilizing this architecture, numerous skin disease classification and segmentation methods have been proposed by various researchers, such as Liu et al. (12), who proposed a multi-knowledge-learning-based system that consist of dual-branch encoder–decoder network including a lesion-area learning and feature co-learning for segmentation of cutaneous T-cell lymphomas. The first component, Lesion Area Learning Module, creates an attention map with a lesion edge feature and produces a binary segment result. The Feature Co-learning module provides attention maps for each branch. Similarly, Zhang et al. (13) introduced a metric-inspired Kappa loss function and implemented it in a modified U-net for skin segmentation lesions. Experimental results demonstrate that the CNN trained with the proposed loss function outperformed a similar architecture with Dice loss. Unlike Dice loss, Kappa loss considers all pixels in the image, including the true negative that enhances the accuracy against ground truth predictions. These studies highlight the importance of loss function and attention modules with CNN’s backpropagation in improving accurate lesion identification and segmentation.

Beyond segmentation, several CNN-based deep learning architectures and ensemble learners have been proposed for accurate lesion classification. Ali et al. (14) introduced a model that evaluates several pre-trained models VGG-16, ResNet50, and InceptionV3 on MSLD dataset, which includes 228 images and three types of skin lesions. Moreover, data augmentation is applied to expand the sample size with threefold cross-validation. Among all evaluated models, ResNet50 achieved the highest accuracy of 82.96%. In another study, A. N. Murthy et al. (15), presented a novel approach using kernel-weighted fuzzy local features C-means clustering technique (K-FCM) for segmentation. Additionally, long short-term memory (DLTM) and the tunicate group algorithm (TSA) are used to detect skin illnesses and categorize normal and abnormal classes. The proposed DLSTM-TSA approach performed well, with an accuracy of 99.34% on small binary dataset. Similarly, J. Singh et al. (16) developed a system by integrating metaheuristic optimizers with different AI-based classification algorithms. To further understand the patterns, numerical features were also retrieved based on different parameters such as mean, area, size in aspect ratio, and width. The model achieved 87.30% accuracy when optimized with random search cross-validation and 90.56% with Bayesian search cross-validation and attained 74.89% without changing the hyperparameters. In another study by Y. Jusman et al. (17), Bespoke CNN, VGG-16, and Multi-layer Perceptron are proposed for skin cancer classification on the HAM10000 dataset. The efficacy of each trained model is then evaluated for accuracy and computing duration for classification. Further experimental configurations validate the effectiveness of the pre-trained deep learning model compared to other techniques for skin disease detection. Likhitha et al. (18) employed CNN and SVM for classification and compared the experimental results that highlight how CNN algorithm performs well and achieves an accuracy of 95.03% compared to SVM that achieves 93.04% accuracy. These results enhance the confidence that CNN algorithms work more accurately than other SVM algorithms. Barbedkar et al. (19) proposed a bidirectional convolutional U-Net framework for segmentation and DenseNet and VGG-19 for classification. The experimental results demonstrate a skin lesion segmentation performance with 83.09% IOU and 90.66% coefficient. Furthermore, the VGG-19 model achieved an accuracy of 97.29%, which is significantly higher compared to DenseNet, highlighting its superior ability to classify skin lesions. Ahmed et al. (20) proposed a fine-tuned Skin Cancer Classification Network (SCCNet) that integrates the Xception model with four distinct fine-tuned layers. The proposed model was trained on the publicly available ISIC-2018 skin cancer dataset and accurately classified seven different classes. The proposed SCCNet model outperformed other contemporary methods with an outstanding 95.20% accuracy, 95.14% precision, 95.00% recall, and 95.14% F1-score in terms of classification accuracy for various skin cancer types.

The evolution of these CNN-based deep models significantly improved the classification and segmentation of skin lesions. However, these models still depend mainly on visual features that make them sensitive to data imbalance and are less effective for unseen lesion types. Therefore, there is a need for more context-aware architecture, such as transformers, that emerged as an alternative to conventional CNNs. Their self-attention layer has the ability to capture long-range dependence where CNNs struggle. Dosovitskiy et al. (21) introduced the transformer based on this concept; several studies adapted transformers in dermatology. Guang Yang et al. (22) proposed a transformer encoder architecture that consists of N identical layers, and each further comprises two sublayers. The first sublayer captures long-range dependencies through a multi-head self-attention mechanism, and the second layer performs feature transformation and non-linearity by utilizing a fully connected feed-forward network. Moreover, layer normalization is applied on both sublayers to find out residual connections. To enhance generalization, after the encoder, a classification block is introduced, which is integrated with batch normalization. For general visual representation, transfer learning is applied on ImageNet dataset and, for specialized dermoscopic fine-tuning, is conducted on HAM10000. The experimental results demonstrate that the proposed model outperforms the Inception-ResNet-v2 model with an accuracy of 94.1%. Similarly, Rongtao Xu et al. (23) introduced a transformer-based architecture (SkinFormer) that fuses and captures texture information for skin lesion segmentation. Specifically, the Kurtosis-guided Statistical Counting Operator (KSCO) mechanism is designed to quantify the texture features extracted by the model. Furthermore, there are two modules: the Statistical Texture Transformer (STF-Trans) for structural and statistical feature fusion and the Statistical Texture Enhancement Transformer (STE-trans) for defining lesion boundary. A Dice score of 93.2% was obtained on ISIC 2018, showing the model’s extensibility.

Although transformers demonstrate high feature transferability, their implementation relies on visual data and they are limited in capturing descriptive information, which highlight the need for hybrid models. Building on these findings, Wang et al. (24) introduced an interpretability-based multi-model CNN (IM-CNN) capable of multiple-class classification using skin lesion images and patient information for skin cancer identification. The investigation demonstrates that the suggested method significantly outperforms well-known deep learning architectures, such as DenseNet and ResNet, in melanoma identification. Specifically, the multi-model approach achieves an average increase of 21% in AUC_SEN_80 and 72% in sensitivity. Furthermore, a co-attention-based adaptive multi-modal fusion (AMF) method was presented by Li et al. (25) for treating dermatosis. This technique aligns and fuses images and metadata by evaluating their optimization difference using the Euclidean norm, using the co-attention approach. The PDA-UFES-20, ISIC 2019, and HAM10000 datasets were used to evaluate the effectiveness of the suggested method. The results demonstrate that the recommended methodology performed better than the alternative methods. Additionally, another co-attention fusion network (CAFNet) was used by He et al. (26), which extracts clinical and dermoscopic image features using a hyper-branch architecture that fuses optimized features at the early network stages. Each CAF module’s co-attention (CA) block integrates two modalities using a cross-model attention approach. An attention fusion (AF) block builds a fine-grained fusion network by dynamically determining the fusion ratios for pixel-wise multi-model integration following the CA block. A deep-supervised loss with an integrated prediction technique is recommended for improved prediction. CAFNet outperforms the state-of-the-art algorithms with an accuracy of 76.8% on the seven-point assessment datasets.

Prior research highlights that the hybrid models contribute to improve diagnostic accuracy, but their lack of interpretability motivates the integration of explainable AI (XAI) with deep models. Furthermore, these XAI tools integrated with hybrid models not only increase diagnostic confidence but also enable clinicians to perform in-depth image analysis rather than a simple visual inspection. This detailed analysis creates a more interactive and informative diagnostic process that significantly improves the quality of patient care. Similarly, VQA-based is introduced by Zhou et al. (27) for skin cancer diagnosis, which integrates visual and textual data. This novel technique, termed Mutual Attention Transformer (MAT), consists of guided-attention and self-attention blocks to facilitate concurrent interactions between information from both modalities. Moreover, a fusion mechanism is integrated with represented properties before reaching the final output layer of classification. The experimental results on the HAM10000 dataset demonstrate that the proposed method outperforms current state-of-the-art methods for diagnosing skin cancer.

Despite these advances, only a few approaches integrate images and domain-specific lesion-based descriptions for early-stage disease diagnosis across diverse skin types. While existing models outperformed well, they often rely on image-only deep learning models that are mostly dependent on visual patterns, which is critical in medical domain. These image-only models show high performance that is not useful while taking critical decisions. Moreover, medical imaging analysis is resource intensive and requires interactive and comprehensive systems that capture clinically meaningful attributes, such as lesion border, color variation, asymmetry, regularity, precise location, and patient age. These attributes support the selection of appropriate clinical procedures and assist dermatologists in making better decisions. To address this limitation, a descriptor-guided multi-modal framework is introduced, which integrates visual features with structured lesion descriptors derived from established dermatological criteria. These integrated features enable clinicians to analyze complex skin lesions, describe diagnostic outcomes, and make more accurate decisions. Moreover, cross-modal attention is employed to effectively align semantic and visual feature representations, improving diagnostic performance, feature learning, and model interpretability.

## Lesion descriptor generation and dataset overview

To evaluate multimodal diagnostic frameworks, a dermoscopic dataset is required; there are many different publicly available datasets, such as HAM10000 (28) and ISIC 2018 (29). However, these datasets primarily contain dermoscopic images without structured clinical metadata. To fulfil this gap, a newly structured descriptor dataset is constructed by extracting lesion-specific attributes from images using dermatological criteria that provide domain-informed complementary semantic representation for model learning. For that purpose, the publicly available ISIC 2018 (30) was used, which provides access for research and educational purposes under non-commercial terms and conditions (31). Dermoscopic images were obtained from ISIC 2018, which consist of a collection of different skin lesion images with varying pigmentation and morphological structures. A total of 10,015 images were carefully selected, which cover all classes of ISIC 2018. The class distribution of this dataset is illustrated in **Table 1**.

**Table 1:** Class distribution of the ISIC 2018 dataset.

Class	Number of images
Melanocytic nevus (NV)	6,705
Melanoma (MEL)	1,113
Benign keratosis-like lesions (BKL)	1,099
Basal cell carcinoma (BCC)	514
Actinic keratoses (AKIEC)	327
Vascular lesions (VASC)	142
Dermatofibroma (DF)	115
Total	10,015

Each class contains a different number of images that exhibit noticeable class imbalance. This class imbalance is effectively addressed during training and experimental setup. Additionally, through a multi-stage processing pipeline, structured lesion descriptors are generated for each image. First, images are preprocessed and skin lesions are automatically isolated using Segment Anything Model (SAM), which ensures that only clinically relevant features are processed by removing irrelevant background regions. We employed SAM for lesion segmentation on the ISIC 2018 dataset because it achieved a Dice score of 94.25% and an IoU of 92.81% against the official ground-truth masks that demonstrate its effectiveness for accurate segmentation. Furthermore, these extracted lesions $I_{lesion}$ are normalized and resized before processing using a visual encoder. In the second step, each lesion is analyzed using ABCD Rule (32), that is, the four-stage diagnostic criteria for lesion evaluation: (i) asymmetry (A) that evaluates lesion shape and size, (ii) border irregularity (B) that examines the edges on skin lesions and surface roughness, (iii) color variegation (C) to analyze the effect of the non-uniform color distribution and redness level based on pixel intensity, and (iv) diameter (D) to measure the area and perimeters of skin lesions. The description generation process is fully automated and extracts clinically relevant information. Each attribute is determined based on predefined formulas applied on segmented lesion marks. The detailed procedure is presented in **Algorithm 1**.

Algorithm 1

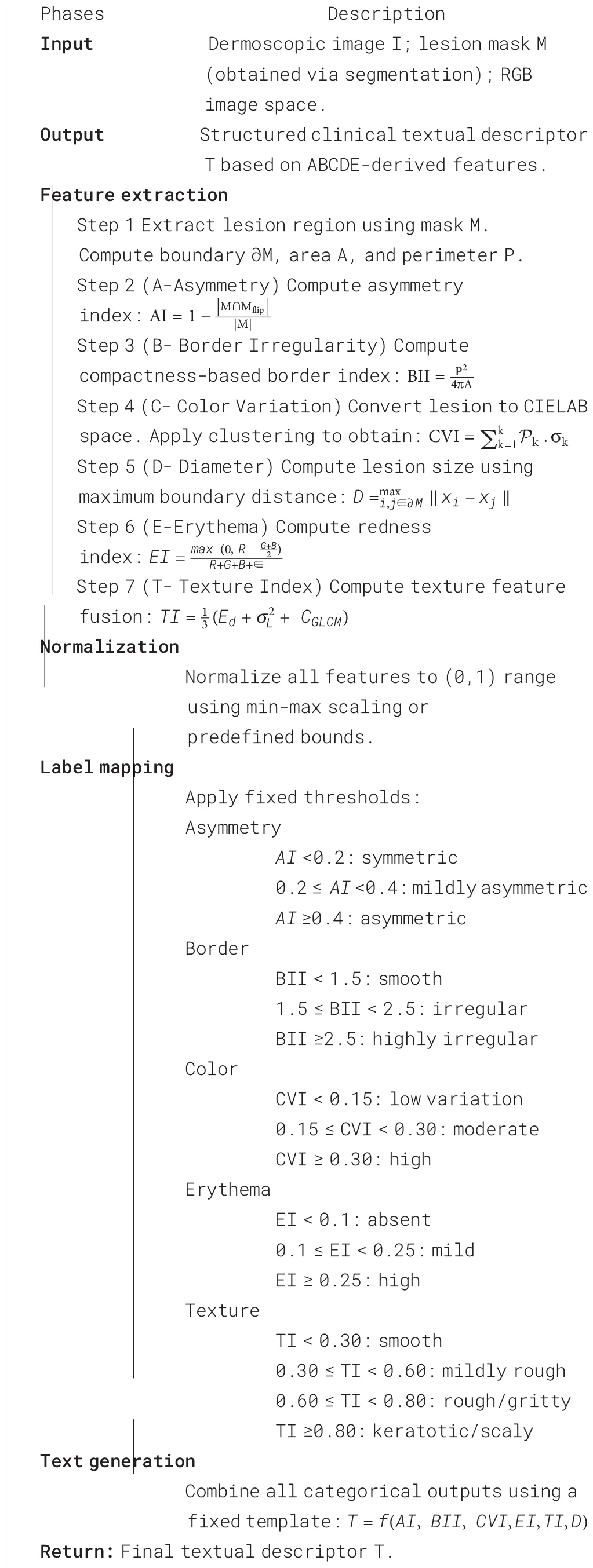

All of the extracted attributes according to their image path, localization, and class labels are organized into structured semantic descriptors and saved in a.CSV file. These descriptors are derived from lesion attributes and provide domain-informed complementary features that capture clinically relevant lesion characteristics rather than functioning as an independent modality. Although the structured descriptors are derived from the same dermoscopic images, this information describes lesion characteristics rather than just simply duplicating pixel-level visual features. Moreover, cross-modal attention is applied to align visual and semantic representations that improve and enhance feature learning, robustness, and interpretability.

Furthermore, these standardized, domain-informed complementary feature representations reduce variability compared with free-form clinical text. Both the images and their corresponding structured descriptors are used jointly for evaluation and training purposes. **Table 2** illustrates examples of some diseases according to their structured descriptors.

**Table 2:** Sample set of diseases and corresponding symptoms.

Image	Description	Label and names
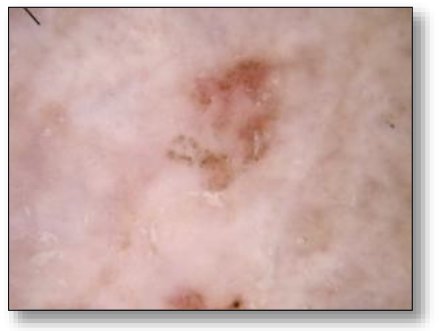	Examination of the lower extremity shows a gray, coarse lesion, with medium-sized and irregular borders and ~25,455 pX² area, ~638.2 px perimeter, and non-erythematous	AKIEC Actinic keratoses and intraepithelial carcinoma
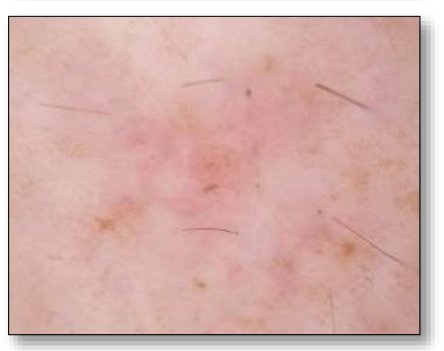	A gritty, ashen lesion is identified on the upper limb, featuring without visible redness and ~106,164 px² area, ~1400.0 px perimeter, large in size, and with irregular borders	BCC Basal cell carcinoma
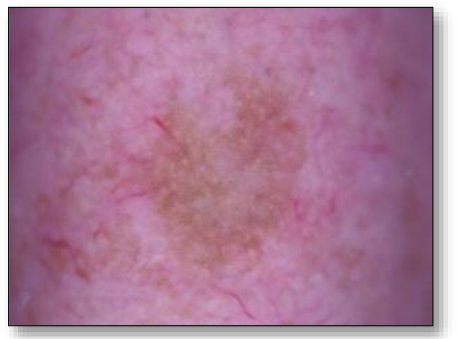	On the unspecified site, a gray, coarse skin lesion is apparent, with mildly irregular borders, large-sized, ~61,062 px² area, ~975.9 px perimeter, and no erythema	BKL Benign keratosis-like lesions
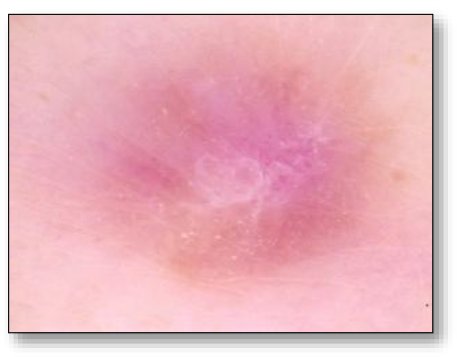	In the region of the lower extremity, there is a mildly rough, gray plaque, featuring ~118,811 px² area, ~1289.1 px perimeter, non-erythematous, large in size, and with well-circumscribed borders	DF Dermatofibroma
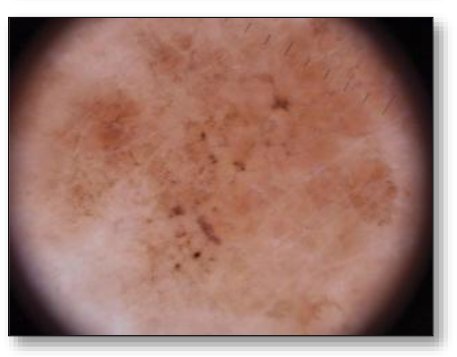	There is a dark brown, coarse skin lesion on the unspecified site with non-erythematous, large, and irregular borders, with ~45,087 px² area and ~867.6 px perimeter	MEL Melanoma
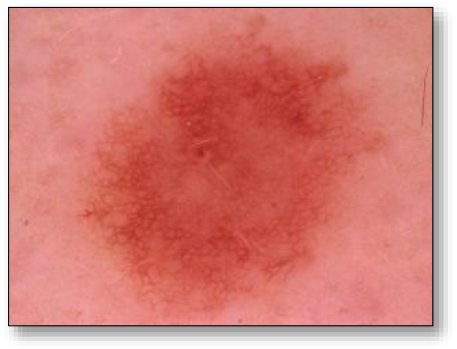	At the unspecified site, we observe an uneven, variegated lesion, displaying without visible redness and ill-defined borders, with ~119,633 px² area and ~146.0 px perimeter, large in size	NV Melanocytic nevus
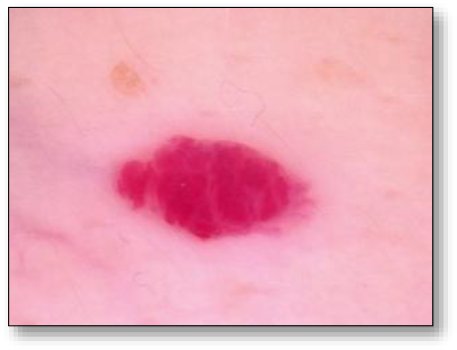	Dermatology exam: An uneven brick-red skin lesion on the unspecified location, with irregular borders and medium in size, with severe erythema, ~32,001 px² area, ~730.2 px perimeter	VASC Vascular lesions
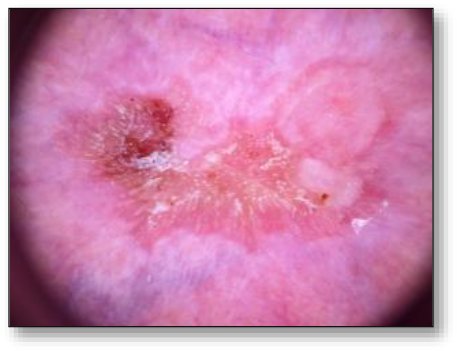	There is a light-brown uneven lesion on the unspecified site, with ~107,685 px² area and ~1367.0 px perimeter, large in size, mild erythema, and ill-defined borders	AKIEC Actinic keratoses and intraepithelial carcinoma
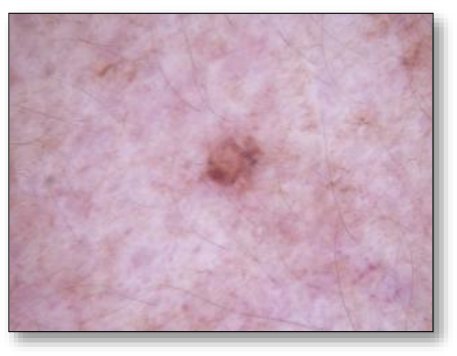	A pale color, uneven patch on the unspecified location, featuring a small size, non-erythematous, ~10,081 px² area and ~379.6 px perimeter, with a smooth border noted on the face	BCC Basal cell carcinoma

Moreover, to ensure performance, a stratified fivefold cross-validation is applied, where the dataset is divided into five equal subsets. These five folds are utilized in such a way that four subsets (80%) of data are used to train the model and one subset (20%) of data is used to validate the model in each iteration. This process is repeated five times with different splits, and these splits were stratified to handle class distribution across folds. The reported performance presents the average experimental results across five stratified cross-validation folds, which preserve class distribution in each fold and ensure unbiased evaluation. To avoid data leakage, during cross-validation, images from the same sample are kept within a single fold, which prevents overlapping between both the training and the validation sets. Furthermore, the structured lesion descriptors and the segmentation output linked with their respective dermoscopic image in the same cross-validation folds during training and validation, which avoids information leakage between the training and validation sets. Additionally, a class-weighted strategy based on inverse frequency is applied in the training set. In this approach, weights are computed for each class to handle class imbalance by assigning higher weights to minority classes, which increased their importance during training. This improves the model’s ability to capture more features from minority classes.

All of the experiments are implemented using PyTorch. Furthermore, the proposed model is constructed by integrating a visual encoder based on the pre-trained ViT-Base/16 with a textual encoder that utilizes BERT-base-uncased. The model was optimized with a set of carefully selected hyper-parameters, including AdamW optimizer, with 0.00002 learning rate, 32 batch size, and trained on GPU for 50 training epochs. Moreover, early stopping mechanism is applied when the validation performance fails to improve. All of the experiments were performed on NVIDIA RTX 3080 GPU with 10 GB memory, and the proposed model takes approximately 3.8 h for training and 18 ms inference time per image, which highlight its adaptability for clinical decision making.

## Materials and methods

This section presents a structural-descriptor-based framework for automated skin disease diagnosis. This proposed framework integrates the visual features of dermoscopic images with its corresponding structured lesion descriptors into a unified feature representation for predication. Conventional single-modality deep learning models are limited in their ability to interpret multiple clinically relevant lesion attributes such as lesion color, texture, and morphology.

In contrast, the proposed dual-branch architecture enables cross-modal reasoning between clinical images and their structured lesion descriptors, which enhances the accuracy of skin disease diagnosis and improves diagnostic performance. The architecture consists of three major components. First, a visual feature encoder extracts complex spatial and texture patterns from images using a pre-trained vision transformer (ViT). Second, a descriptor encoder employs a pre-trained BERT to process structural lesion descriptors to get a contextual embedding. Third, a cross-attention-based fusion module is applied, which integrate these two feature sets and enables the model to understand textual lesion descriptions corresponding to their visual feature.

**Figure 1:**
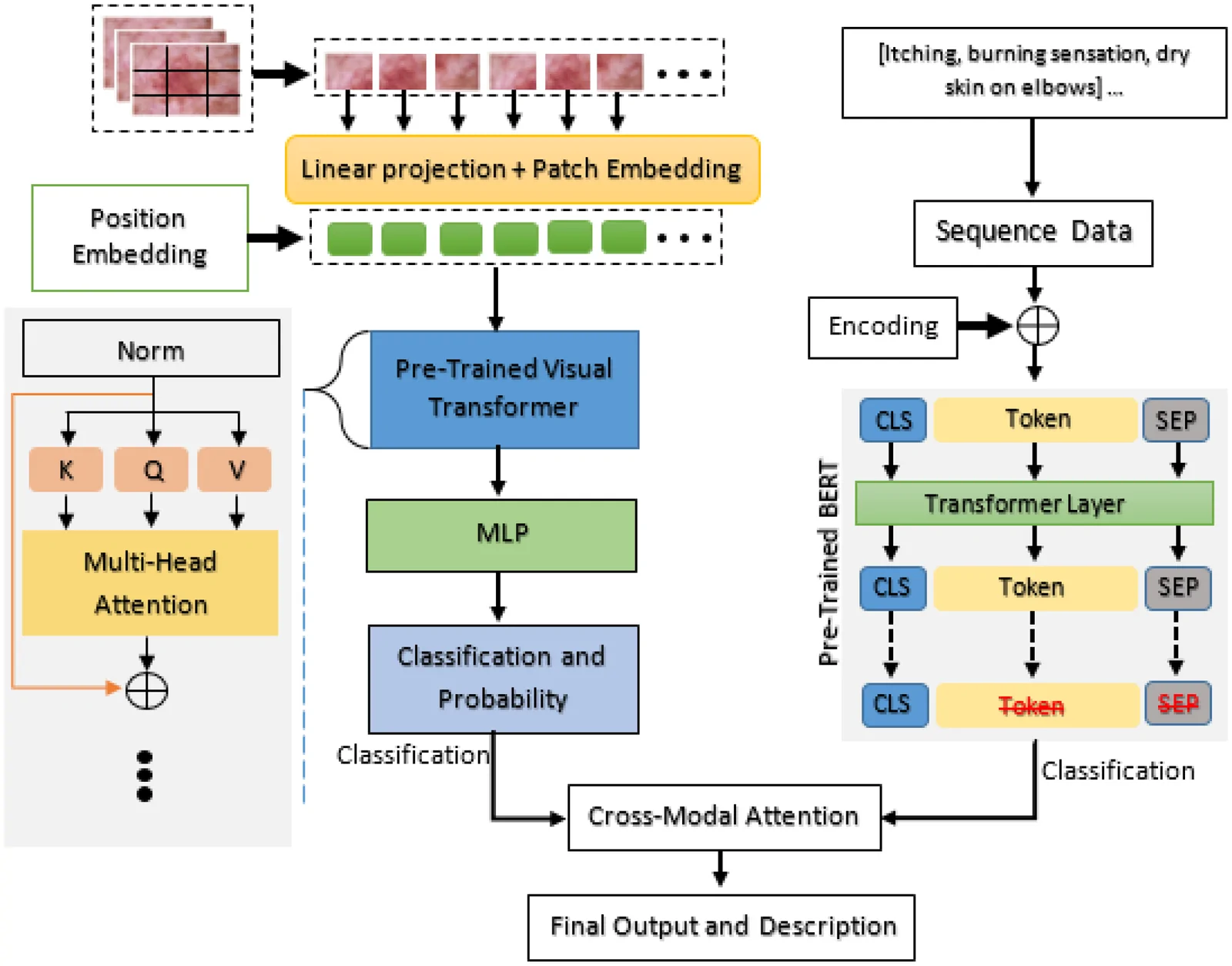
Overview of the proposed skin disease classification model.

The final output is generated by a classification head that identifies different skin diseases based on the descriptor-guided refined visual features. Additionally, to enhance the model’s generalization across diverse skin lesions, the network is optimized through a task-specific loss function. An overview of the proposed skin disease detection model is shown in **Figure 1**, and the notation of symbols used in the proposed framework is also described in **Table 3**. A step-by-step explanation about each component is provided in the following subsections.

**Table 3:** Description of symbols and their notation as used in the proposed model.

Symbol	Description
(I)	Input dermoscopic image
(H,W,C)	Height, width, and channels of input image
$\left(P_{i}\right)$	Image patch extracted from (I)
(N)	Number of image patches
( $X_{img})$	Sequence of image patch embedding
$(E_{pos}$)	Positional encoding vector
( $Z_{img})$	Final visual feature representation (ViT output)
(T)	Sequence of structured lesion descriptor tokens
$(E_{text}$)	Descriptor embedding vector
$\left(Z_{text}\right)$	Final descriptor representation (BERT output)
(Q,K,V)	Query, key, value matrices in attention mechanism
$(\;W_{q}$, $\;W_{k}$, $\;W_{V})$	Learnable projection matrices
$\;(d_{k}$)	Dimension of key vectors (scaling factor)
CMA	Cross-modal attention output
(F)	Fused feature representation
(Y)	Predicted probability distribution over classes
$\hat{(\;Y_{C}}$)	Predicted probability for class (c)
( $Y_{C})$	Ground-truth label for class (c)
$\left(W_{C}\right)$	Class weight for class imbalance handling
C	Total number of classes
L	Final weighted loss function

### Visual encoder

Convolutional neural networks (CNNs) are widely used in dermoscopic imaging and effectively extract texture features and capture local patterns, which are helpful for disease detection and classification. However, these models often struggle to capture global context information and long-range dependencies. To address this, Vision Transformer (ViT) (21) is employed, which is based on a self-attention mechanism with ViT-Base/16 visual encoder.

Unlike traditional models that process directly on pixel values, ViTs first divide $I_{lesion\;}\in {\mathbb{R}}^{H\;\times W\;\times C}$ into fixed-size, small, N non-overlapping patches $\{P_{1},P_{2},.\;.\;.,P_{N}\}c$ each of size P×P. The ViT flattened each patch $P_{i}\;$into one-dimensional vector. Each patch is linearly projected using bias $b_{E}\;$and learnable projection matrix $W_{E}\;$into D-dimensional embedding that serves as “visual token.” Then, these tokens are processed in sequence as shown in Equation 1. This step transforms local image pixel into compact visual tokens that encode relevant features such as texture, color, border irregularity, redness level, and size for skin lesion analysis.

(1) $$X_{img}=[x_{1},x_{2},.\;.\;.,x_{N}]=W_{E}.\;Flatten\;(P_{i})+\;b_{E}$$

where $x_{i\;}\in {\mathbb{R}}^{D}$ denotes embedding of $i^{th}$ patch and $X_{img}\;$is the sequence of embedding patch vector that acts as *visual token*, allowing a transformer to process an image as a sequence and helping to capture fine-grained details within small regions with skin lesions. Moreover, to retain the spatial location of each token in the image sequence, positional embedding is added as shown in Equation 2:

(2) $$X_{img-pos}=X_{img}+PositionalEmbedding(X_{img})$$

In a position-aware patch embedding ( $X_{img-pos}$), a $PositionalEmbedding(X_{img})$ and a special learnable classification [CLS] token are added to each patch token to retain the spatial data and to aggregate global image feature across all patches.

Furthermore, the position-aware sequence $X_{img-pos}\;$is then passed through L transformer encoder layers. A transformer encoder contains multi-head self-attention (MSA) at each layer, allowing every patch token to interact with all others. The mechanism is defined in **Equation 3** as follows:

(3) $$A(Q_{i},K_{i},V_{i})=softmax(\frac{QK_{i}^{T}}{\sqrt{d_{k}}})V$$

where $(Q_{t},K_{t},V_{t})\;$represent query, key, and value metrics derived from input tokens, and A is the attention scores for each token pair in a sequence. Here $QK_{t}^{T}$ is the dot product of the query ( $Q_{t}$) and key ( $K_{t}$) matrices and $V_{t}$ for vector, scaled by the square root of the key’s dimension ( $\sqrt{d_{k}}$), yielding the attention score. Furthermore, a softmax layer is applied to normalize the attention score. The output of MSA is refined by a feed-forward (FFN) multi-perceptron (MLP) as defined in Equation 4:

(4) $$\text{MLP}(X_{img})={\text{FC}}_{2}(\text{σ}({\text{FC}}_{1}(X_{img})))=ReLU(xW_{img\_1}+b_{img\_1})W_{img\_2}+b_{img\_2}$$

where ${\text{FC}}_{1}$and ${\text{FC}}_{2}$ are fully connected layers, σ(·) is a nonlinear activation function applied along with the weight matrices $W_{img\_1}$ and $W_{img\_2}\;$of two fully connected layers, and $b_{img\_2}$ and bias $b_{img\_1}$ are the biases that are followed by a non-linear activation function, ReLU. The input is first transformed by ${\text{FC}}_{1}$and then passed through an activation function and then ${\text{FC}}_{2}$ for feature refinement. Self-attention mechanism captures long-term connections between different image patches. This makes the architecture effective for specific large-scale tasks. Furthermore, this mechanism improves the model’s generalization and reduces dependency on task-specific training. After the final Transformer encoder, the output of the [CLS] token is utilized as the global feature vector for dermoscopic images, as defined in **Equation 5**:

(5) $$Z_{img}=X_{CLS}{\mathbb{R}}^{D_{img}}$$

where Zimg is the final visual, representing embedding of the [CLS] token and indicating the dimensionality of the vector. Furthermore, this representation improves the model’s generalization and reduces dependency on task-specific training.

### Descriptor encoder

In this proposed architecture, textural BERT-base-uncased encoder is employed to process structured lesion descriptors corresponding to dermoscopic images. These lesion descriptor encoders accurately capture relevant attributes that describe lesion appearance such as color, size, location, redness level, border regularity, and surface roughness.

To encode this information, we applied a pre-trained BERT model that transforms the structure lesion descriptor sequences into contextual embedding. The traditional text representation models, such as n-gram and TF-IDF, rely on word frequency and neglect the contextual relationship between descriptor attributes. These approaches focus on local patterns and are limited in understanding long-range dependencies among lesion characteristics. Moreover, BERT captures contextual, bidirectional semantic relationships within descriptor sequences. This helps the model to represent interactions between coherent and contextually relevant lesion attributes with visual features for enhanced classification.

Let the structural lesion descriptor sequence be $T=\{t_{1,}t_{2,}\ldots ,t_{n}\}.\;$Each token is first tokenized and embedded into a dense vector. These embeddings represent each word order in the sequence as shown in Equation 6:

(6) $$E_{text}=\{e_{1,}e_{2,}\ldots \ldots .e_{n}\}$$

where, $e_{i\;}\in {\mathbb{R}}^{D_{text}}$ represent embedding of $i^{th}$ word. Moreover, positional encodings $E_{pos}$ are added to the embedding vector to preserve word order as defined in Equation 7:

(7) $$X_{text-pos}=E_{text}+E_{pos}$$

where $E_{pos}$ represent learnable position embeddings. The BERT model consists of multiple transformer encoder layers $L_{t}$ that processed this representation. Each layer comprises a self-attention mechanism (MSA) and feed-forward networks (FNNs). Input_Embedding is then passed through these layers as expressed in Equation 8:

(8) $$H_{i}=MHSA(H_{i-1})+\;FFN(H_{i-1})$$

where $H_{i}$ represents the output feature matrix of $i^{th}$ BERT encoder layer, and $H_{i-1}\;$represents the input embedding from previous layers. In the last layer, tokens such as [SEP] are excluded because they are not required for downstream textual representation; only [CLS] tokens are retained because they are required for further sentence-level embedding and input description processing. After that, final embedding is represented as in Equation 9:

(9) $$Z_{text}=X_{CLS}{\mathbb{R}}^{D_{text}}$$

This representation $Z_{text}$ captures the interaction among all lesion attributes in a compact form, which helps in its effective integration with visual features.

### Cross-attention fusion module

In this step, feature fusion is performed through a cross-attention mechanism that results in integrated feature representation for classification. In this fusion mechanism, structured descriptor lesion attributes guide further refinement of visual features in model prediction. To capture these inter-dependencies between visual features and structured descriptors, a cross-model attention is applied. The visual embedding obtained from vision transformer represented as $Z_{img}\in {\mathbb{R}}^{D_{img}}$ is used as query, ( $Q_{image}$), and textual descriptor embedding represented as $Z_{text}\in {\mathbb{R}}^{D_{text}}$ is used as key, $(Key_{text}),$ and value, $Value_{text}$, as defined in Equation 10:

(10) $$Z_{fusion}=Softmax(\frac{\left(Z_{img}\;W_{q}){\left(Z_{text}\;W_{k}\right)}^{T}\right)}{\sqrt{d_{k}}})(Z_{text}\;W_{v})$$

where CMA is the cross-model attention that estimates the image features $Q_{image}\;$using a learned weight matrix $W_{q}\;$and the textual features $K_{text}$ using $W_{k}$. Then, the attention scores are computed, and Softmax is used to normalize these scores into probabilities from the resulting scores. The process above helps to align and learn inter-dependencies between visual features with their corresponding lesion descriptor attributes. This attention-enhanced feature representation captures how these descriptors take part in the refinement of visual features. Furthermore, this fused representation is processed by the classifier head, which also generates the final prediction probabilities described in Equation 11:

(11) $$\hat{Y}=Softmax(W_{out}\cdot Z_{fusion}+b_{out})$$

where $\hat{Y\;}=[\hat{Y_{1}},\hat{Y_{2}},\ldots ,\hat{Y_{C}}]$ represents the predicted class probability distributions over skin lesion categories C. After the final output, a linear transformation is applied using the weight matrix $W_{out}$ and bias $b_{out}$, followed by Softmax, that transforms the output into an expected distribution across possible outcomes. Overall, the procedure for this process is summarized in **Algorithm 2**, which shows the effective integration of relevant structural lesion descriptors with associated visual features using cross-attention-based fusion to produce cohesive and diagnostically meaningful predictions.

Algorithm 2

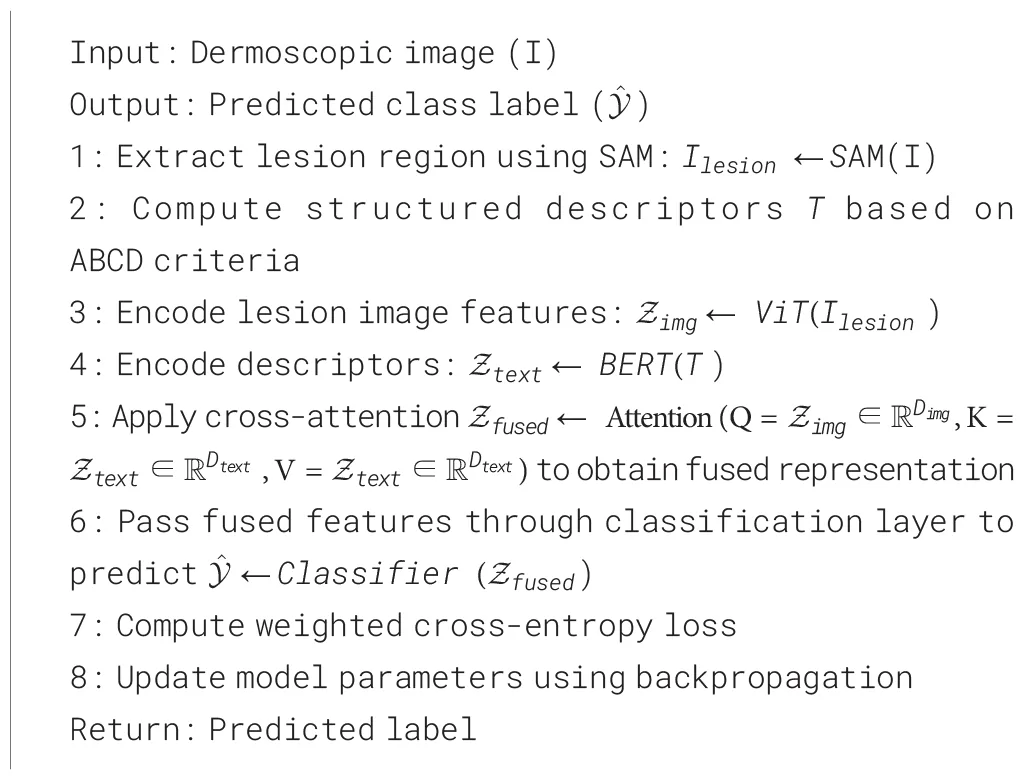

### Training and optimization for class imbalance

The dataset ISIC 2018 used in this proposed work exhibits class imbalance among different lesion classes, which can hinder efficient learning from minority classes. To mitigate this, a weighted cross-entropy loss is applied. During training, class weights are computed based on inverse frequency, assigning higher importance to minority classes, as shown in Equation 12:

(12) $$w_{c}=\frac{N}{C.n_{c}}$$

Here ${\text{w}}_{\text{c}}$ is the class-specific weight, and N represents the total number of samples, whereas the total number of classes is represented by C, and the number of samples per class is represented by ${\text{n}}_{\text{c}}$. Additionally, the final loss is defined as follows: model parameters are optimized using task-specific loss functions as defined in Equation 13:

(13) $$L\;=\;-\sum_{C=1}^{C}w_{c}Y_{C}\;log(\hat{Y_{C}})$$

Where $Y_{C}$ represents ground-truth label, $\hat{\;Y_{C}}$ defines the predicted class probability, and $w_{c}$ is the class-specific weight. Overall, this strategy improves the model’s ability and ensures the contribution of minority classes in the optimization process.

## Results and analysis

To evaluate the effectiveness of the proposed model, a series of comprehensive experiments were conducted on multimodal skin disease datasets. These experiments highlight the performance of the proposed model using visual and structured descriptor features as well as their fusion guided by structured lesion description. Since a specific dataset for multi-modal skin disease classification is not publicly available, the dataset’s characteristics, including its structure and features, are systematically explored.

In the first experiment, the effectiveness of individual modalities is analyzed in disease prediction. The model is trained and tested separately using image-only inputs and structured descriptor-only inputs. Additionally, the proposed study also investigates the effectiveness of individual CNN models in skin disease prediction. This assesses how well the model performs with each data type independently. Overall, experimental results on image and structured descriptor data are defined in **Table 4**.

**Table 4:** Overall performance comparison across image-only, structural descriptor, and fusion-based model integration.

Model configuration	Input type	Accuracy (%)	F1-score (%)	MCC	Cohen’s kappa	Footnotes
BERT-base-uncased (descriptor only)	Structured lesion descriptions	88.42	87.95	0.85	0.86	Fine-tuned using structured descriptors derived from ABCD criteria
CNN (image only)	Skin images	89.76	89.21	0.87	0.88	CNN trained using dermoscopic images
Image + descriptors (no attention)	Image + structured descriptors	91.12	90.43	0.89	0.90	Direct concatenated without features alignment
Image + descriptors + (fusion with CMA)	Image + structured descriptors	93.54	93.01	0.92	0.93	Combined features using ensemble strategy without cross-attention
Proposed framework (cross-attention integration)	Image + structured descriptors	96.87	96.32	0.95	0.97	Descriptor-enhanced transformer model with cross-attention alignment

The experimental results highlight that image-only models perform well for skin disease classification. Moreover, its integration of structured lesion descriptor improvement can be observed across all configurations. It is important to note that, without attention mechanisms, simply integrating images and structured descriptors leads to an enhanced accuracy of 91.12%, which indicates that additional structure descriptors are not just redundant information. Furthermore, addition of cross-attention improved the accuracy up to 96.87%, which indicates that aligning visual features with structured descriptors improved the model in capturing clinically relevant patterns.

Another experiment is conducted to evaluate the performance of visual features (image) and textual structured lesion descriptors. In this experiment, accuracy and precision are achieved by the pre-trained models as demonstrated by their effectiveness in handling these unimodal inputs. Furthermore, this illustrates the effectiveness of the proposed model in handling unimodal inputs, providing a solid baseline for subsequent multi-model data integration. This analysis focuses on the performance of CNN models (AlexNet, VGG16, ResNet18, and ResNet50), ViT branch, and both BERT variants on individual modalities. Each model is evaluated independently to understand its capability before integrating these modalities into complex tasks. **Table 5** shows how well each model performs in disease prediction.

**Table 5:** Comparison of the individual models in disease prediction using a single modality.

Base model	Features	Accuracy (%)	Precision (%)	Recall (%)	F1-score (%)
AlexNet	Visual Features	92.11	92.13	92.11	92.08
VGG16		93.86	92.86	92.85	92.86
ResNet18		94.26	93.27	93.26	93.26
ResNet50		94.74	93.79	93.74	93.73
ViT-Base/16		94.77	94.78	94.77	94.75
Logistic regression	Structured lesion descriptors	62.50	66.82	62.50	61.74
DistilBERT		88.60	87.21	87.44	86.12
BERT-large-cased		85.60	83.40	82.14	82.72
BERT-based-uncased		88.42	87.90	87.80	88.10

### Fusion performance

An experiment was conducted to focus on validating the strength of the proposed cross-attention model. This experiment aimed to capture deeper connections between visual features (from images) and symptom descriptions (from text modalities). **Table 6** further illustrates how well the model integrates and balances information from both sources to improve disease prediction. The results table demonstrates that the proposed model significantly outperformed across all evaluation metrics with 96.87% accuracy.

**Table 6:** Performance comparison of feature combinations.

Metric	Image features	Text features	Multimodal (image + text)	Improvement (multimodal vs. best unimodal)
Accuracy (%)	94.77	85.6	96.87	+2.10%
Precision (%)	94.78	83.4	96.62	+1.84%
Recall (%)	94.77	82.1	96.79	+2.02%
F1-score (%)	94.75	82.7	96.52	+1.77%

The performance of the proposed model for each class is reported in **Table 7**, generating enhanced results across all classes. The majority class (NV) exhibits the highest performance, as expected, with minority classes (AKIEC and DF) showing lower recall performance due to class imbalance. However, this is handled by using a weighted cross-entropy loss, which facilitates learning of minority classes. The model maintains a balance between precision and recall for all classes, demonstrating generalization. Furthermore, the area under the curve (AUC) is provided to assess discrimination ability. The high AUC scores also indicate enhanced separability for both majority and minority classes, indicating the effectiveness of the proposed cross-attention-based approach.

**Table 7:** Performance comparison of feature combinations.

Class	Precision (%)	Recall (%)	F1-score (%)	AUC
Melanoma	96.2	95.8	96.0	0.98
Melanocytic nevus	97.8	98.1	97.9	0.99
Basal cell carcinoma	96.1	95.5	95.8	0.97
Actinic keratoses and intraepithelial carcinoma	94.8	93.9	94.3	0.96
Benign keratosis-like lesions	96.0	95.6	95.8	0.97
Dermatofibroma	95.2	94.0	94.6	0.96
Vascular lesions	96.0	95.0	95.5	0.98

To further evaluate the performance of the proposed approach, the model uses symptom descriptions and corresponding skin images to identify diseases such as eczema, melanoma, psoriasis, and acne. The model provides a diagnosis for each condition with a high confidence level ranging from 76% to 93%, as shown in **Table 8**. This highlights the model’s reliability and its potential to accurately identify skin diseases based on text-based and visual features.

**Table 8:** Qualitative analysis of case-based model predictions and corresponding features.

Symptom description	Image	Model-identified disease	Confidence (%)	Matched features	Correct/incorrect
A scaly, dark-colored skin lesion is appreciated on the unspecified site, showing irregular borders, pronounced redness, ~129,714 px² area, ~1587.8 px perimeter, and large size	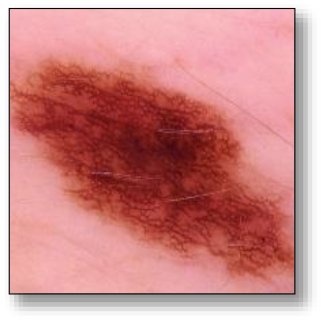	Melanocytic nevus	90	Dark color, irregular border, large size	Correct
A rough, pinkish plaque is appreciated on the unspecified site, with ~121,270 px² area, ~1285.5 px perimeter, irregular borders, severe erythema, and large size	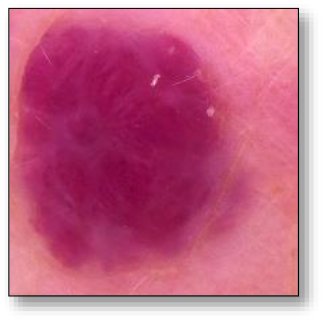	Actinic keratoses and intraepithelial carcinoma	31	Brick-red coloration, gritty coarse surface, large lesion size, severe erythema	Incorrect
Findings at the lower extremity: A dark brown, gritty asymmetric patch, showing non-erythema and measuring as large, with ~40,202 px² area, ~765.3 px perimeter, and well-circumscribed borders	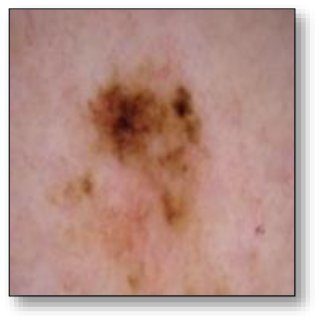	Melanoma	93	Dark brown pigmentation, rough surface, Asymmetry shape, no erythema (non-inflamed), circumscribed borders	Correct
Examination of the unspecified site reveals a pinkish uneven skin lesion, showing moderate erythema, ~100,023 px² area, ~1,407.4 px perimeter, and large size with irregular borders	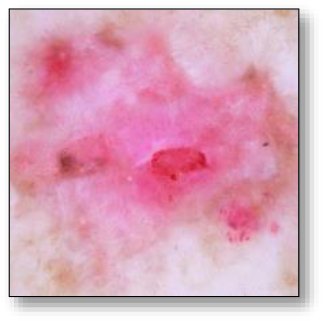	Actinic keratoses and intraepithelial carcinoma	87	Pinkish coloration, uneven surface texture, moderate erythema, large lesion size, irregular borders	Correct
A multicolored gritty patch is appreciated on the upper limb, exhibiting large size, ~100,845 px² area, ~1,267.3 px perimeter, irregular borders, and moderate erythema	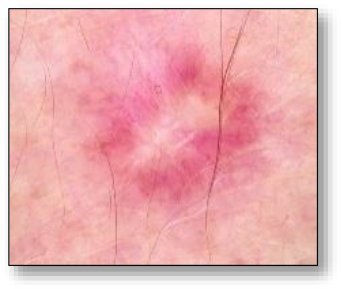	Dermatofibroma	91	Multicolored pigmented patch, gritty surface, large lesion size, irregular borders, moderate erythema (redness)	Correct
Examination of the unspecified site shows a gray, gritty lesion, featuring large size, ~78,332 px² area, ~1,108.1 px perimeter, and mildly irregular borders; non-erythematous	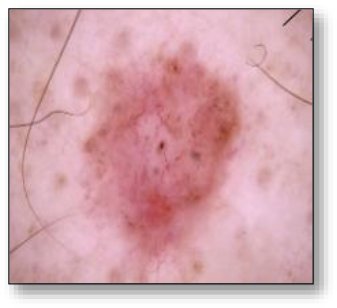	Benign keratosis-like lesions	56	Scaly texture, irregular border, non-inflamed	Incorrect

### Symptom descriptor relevance and interpretability analysis

To assess the strength of the model between text-based lesion symptom descriptors and disease predictions, user-defined inputs were ranked by their similarity to a target disease as shown in **Table 9**. These user-defined symptoms are encoded using textual encoder and then compared with the already learned features. The rankings, ranged from 1 (highest similarity) to 5 (lowest similarity), are computed against disease representations, which helps to identify the most relevant symptoms for an accurate disease classification. A higher rank shows that it has strong correspondence with learned feature representation. Moreover, there are similar classes within the results because of overlapping features across different conditions. This analysis highlights how the model learns clinically meaningful patterns along with raw image features that validate the role of descriptor-guided framework as a decision support system.

**Table 9:** Top five rank results with a similarity score of text-input disease symptoms.

User-defined symptoms	Disease/score similarity				
	Rank 1	Rank 2	Rank 3	Rank 4	Rank 5
Rough gray patch on leg that look dry and scaly	BCC | 0.8341	BCC | 0.8296	BKL | 0.3966	MEL| 0.3531	AKIEC | 0.1578
Multicolored bump that has gritty appearance on my lower leg	NV| 0.8491	NV | 0.8465	MEL | 0.6178	BKL | 0.4890	DF | 0.3732
Dark pigmentation around a previously light mole on the back	MEL | 0.9472	MEL | 0.9352	MEL | 0.9331	MEL | 0.9313	BCC | 0.3531
Small shiny pinkish red bump on nose that have tiny blood vessels	NV | 0.8553	NV | 0.8517	MEL | 0.7175	VASC | 0.4671	BCC | 0.3096
A rough, brick reddish patch has appeared on my abdomen	VASC | 0.942	MEL | 0.6081	NV | 0.5911	DF | 0.3312	BKL | 0.3201
There is a large, irregular multicolor rough lesion appearing on my upper arm	DF | 0.9411	DF | 0.9380	MEL | 0.7098	BKL | 0.5076	AKIEC | 0.5061

**Figure 2:**
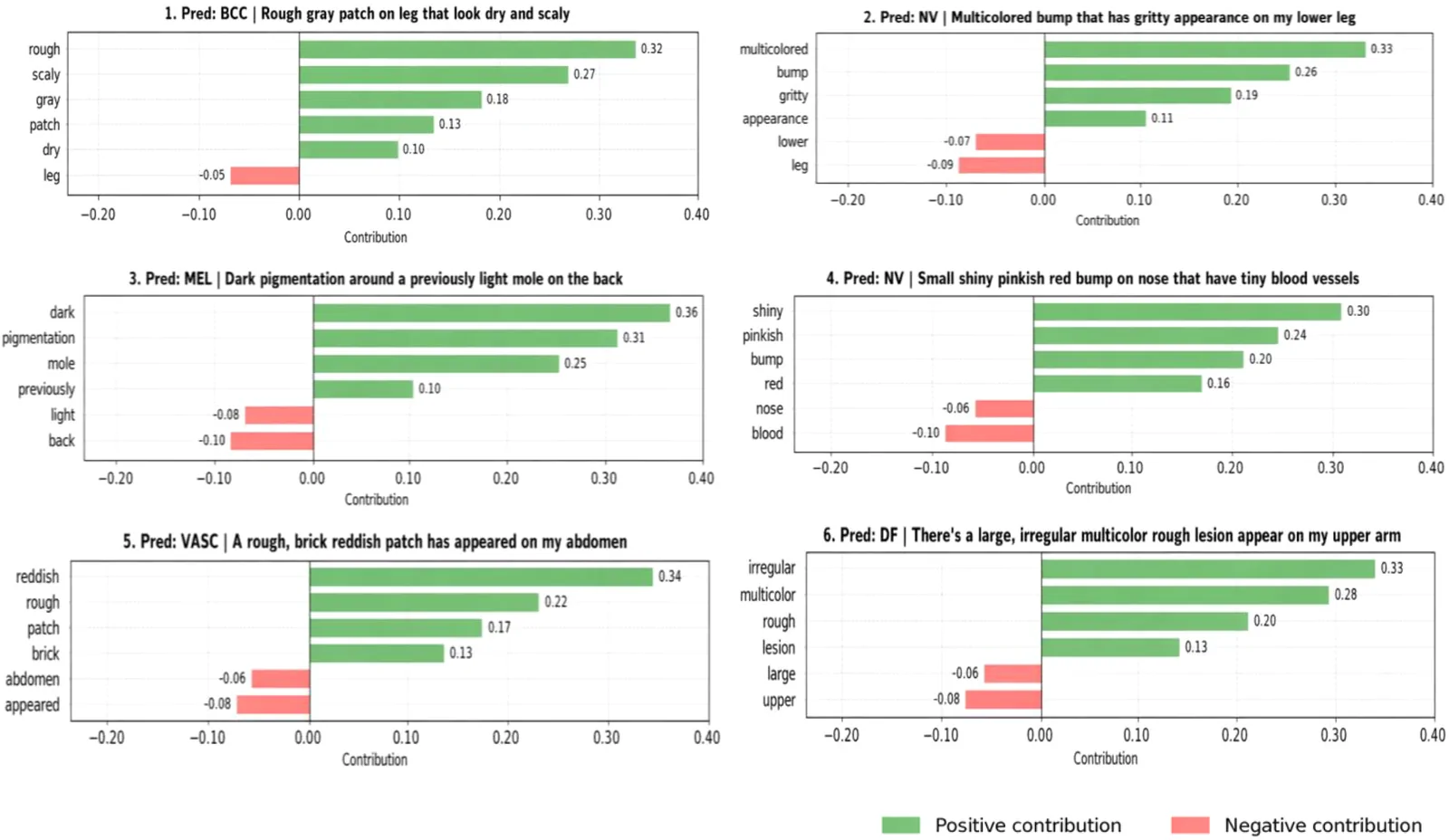
LIME-based explanation for representative skin disease.

In order to enhance interpretability, Local Interpretable Model-Agnostic Explanations (LIME) is applied to understand the role that textual features play in making classification decisions, which are reported in **Figure 2**. The findings show that the model emphasizes more on clinically meaningful descriptors. Such terms as “dark”, “pigmentation”, and “mole” have a powerful impact on melanoma prediction, whereas “rough”, “scaly”, and “dry” are connected with basal cell carcinoma. In the same way, attributes such as “shiny”, “pinkish”, and “bump” are used to predict nevus, meaning that the model was able to identify the relevant semantic patterns.

There were, however, minor inconsistencies, where other class predictions were influenced by the melanoma-related features, including “irregular” and “multicolor”, which create a possibility of bias because of the imbalance of classes and overlap of features. Furthermore, LIME provides enhanced local interpretability by highlighting feature-level contributions, thus making the model more transparent, while clinical validation by a dermatologist ensures its overall reliability in clinical decision-making.

### Impact of loss function on model performance

**Table 10** shows the impact of various loss functions. The baseline cross-entropy loss performs worse as it is biased toward the majority classes in the imbalanced ISIC 2018 dataset. Class-weighted loss enhances performance by giving more weight to the minority classes. The use of focal loss further improves learning by considering hard samples, resulting in better class balance. However, weighted loss function applied with cross-attention delivers the best results, showing that suitable loss functions play a vital role in achieving accurate and reliable classification of imbalanced medical data.

**Table 10:** Comparison of different loss functions and their impact on the performance of the proposed model.

Loss function	Accuracy (%)	Precision (%)	Recall (%)	F1-score (%)	Remarks
Cross-entropy loss	92.84	91.75	90.92	91.30	Baseline performance; biased toward majority classes
Weighted cross-entropy	94.96	94.12	93.88	93.95	Improved handling of class imbalance
Focal loss	95.72	95.10	94.85	94.97	Better focus on hard and minority samples
Dice loss	93.65	92.90	92.11	92.50	Suitable for overlap-based tasks; less effective here
Proposed (weighted + cross-attention)	96.87	96.62	96.79	96.52	Best performance with balanced learning

### Comparison with existing methods

**Table 11** provides a comparative overview of the proposed model and existing approaches. The proposed model demonstrates competitive results with an accuracy of 96% compared to previous research. However, it is important to note that direct comparison is limited due to differences in modality type, as we evaluate both textual descriptors and images.

**Table 11:** Analysis of performance versus other state-of-the-art models.

Ref.	Year	Technique	Model	Dataset	Classes	Modality type	Accuracy (%)
(16)	2024	Skin disease detection and classification	ResNet50, InceptionV3, MobileNetV2	HAM10000	7	Image	90.56
(33)	2024	Deep learning-based skin lesion classification	Inception-ResNet v2 (SA) + EfficientNet-B4	ISIC 2018	7	Image	85.18
(34)	2024	Hybrid CNN-based classification	CNN + DenseNet	HAM10000	7	Image	93.24
(35)	2025	Hybrid deep learning model	VGG-NIN (VGG19 + NIN)	HAM10000	2	Image	90.00
(36)	2026	Deep CNN-based classification	EfficientNet-B0, ResNet-50, DenseNet-169, Inception-v3	ISIC 2018	7	Image	91.84
(37)	2026	Unified supervised + reinforcement learning	ViSurf (vision–language model)	ISIC 2018	7	Image + text	94.70
Proposed		Visual + structured descriptor fusion	ViT + BERT (cross-attention)	ISIC 2018	7	Image + text	96.87

### Ablation study

To evaluate the contribution of each individual component of the proposed model, an ablation study was conducted. In this analysis, components such as cross-attention, BERT, and ViT are excluded or modified to evaluate their effect. The experiment begins with the complete proposed model, followed by iterative modification to provide empirical validation of how each module influences the final prediction. **Table 12** presents the results of the ablation study, which included a step-by-step evaluation. In the first step, the full model with all components is analyzed with no modifications, and it achieved 96.87%. In the next step, when the cross-model attention was removed, performance dropped to 3.45% and accuracy decreases to 93.42%, which highlight the importance of semantic alignment between modalities. When textual input (symptom description) and BERT model were excluded, there is a significant drop of 7.11%, which is a huge impact on accuracy. Similarly, when visual feature is removed, the accuracy drop to 88.42%, which shows that the model relies heavily on both modalities for optimal performance. Finally, when we replace the traditional CNN model with ViT, it led to an improvement in accuracy.

**Table 12:** Ablation study on the proposed multi-model.

Configuration	Visual encoder	Descriptor input	Fusion method	Accuracy (%)	Performance
Proposed model	ViT	Yes	Cross-attention	96.87	**-**
Without weighted loss	ViT	Yes	Cross-attention	94.96	↓ 1.91%
Without cross-attention	ViT	Yes	Feature concatenation	93.42	↓ 3.45%
Element-wise fusion	ViT	Yes	Element-wise addition	92.88	↓ 3.99%
Image only	ViT	No	**-**	89.76	↓ 7.11%
Descriptor only	No	Yes (BERT)	**-**	88.42	↓ 8.45%
CNN backbone	ResNet	Yes	Cross-attention	91.35	↓ 5.52%
Without SAM (full image)	ViT	Yes	Cross-attention	94.95	↓ 1.92%

### Visual explainability analysis using Grad-CAM

**Figure 3:**
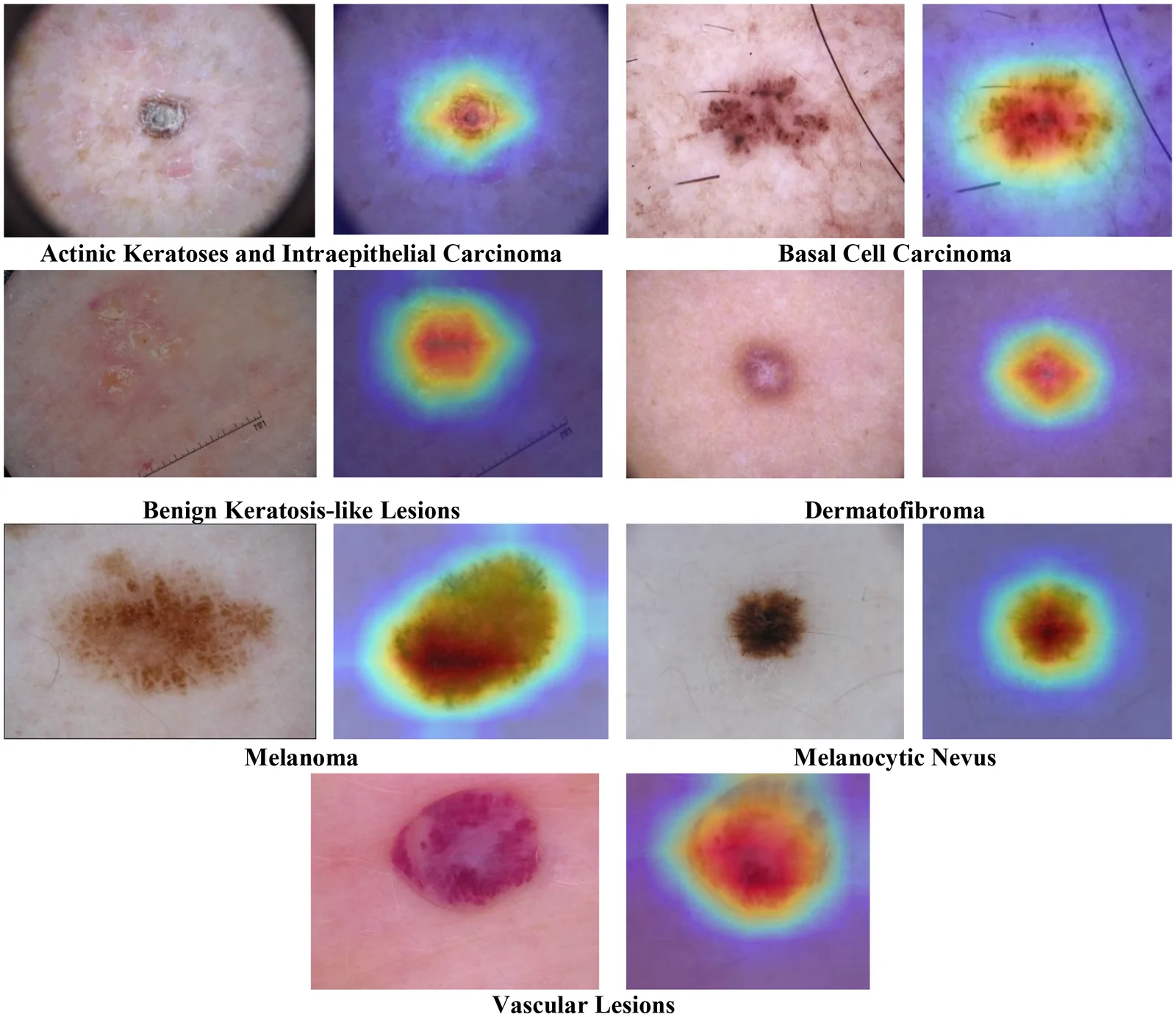
Grad-CAM visualization results for skin lesion classification, highlighting clinically relevant lesion regions identified by the proposed framework.

Grad-CAM is employed, highlighting important lesion features by generating attention heatmaps, enhancing model interpretability, and supporting confident clinical decisions in skin disease diagnosis. To interpret the model predictions, Grad-CAM applies on visual features, which illustrates the strong alignment between the key terms in symptom description and the visual features predicted by the model. A heatmap is generated, as shown in **Figure 3**, which highlights the lesion region where the model detected any irregular borders, pigmentation, inflammation, or other abnormalities. This step also assists dermatologists in making decision and enhancing their confidence due to the visual evidence provided by the model.

These Grad-CAM visualization results highlight that the proposed framework consistently focuses on clinically relevant lesion areas, such as lesion boundaries, pigmentation patterns, and textural abnormalities, enhancing the interpretability of the model’s decision making process.

## Conclusion

Diagnosis of skin disease is a complex challenge around the globe due to many reasons, such as varying skin tones and similar appearance of different conditions, particularly in the early stages. The study addresses the challenge of limited data by combining public image datasets and expert-generated textual datasets to train and test the model. The experimental results show that while visual features alone (using CNNs) achieve accuracy rates of 85% to 93% and transformer-based visual models reach 94.77%, the proposed multi-model cross-attention technique achieves an impressive accuracy of 96.87%. This significant improvement demonstrates the strength of integrating visual features with structured lesion descriptors for robust and accurate skin disease classification. Moreover, deep learning models rely on implicit feature representation, which limits their understandability usually in the medical domain. This approach reduces this by integrating a structured representation that captures lesion-specific properties and provides more clinically meaningful information along with visual features. However, there is a limitation in our proposed framework in such a way that structured lesion descriptors are derived automatically from image-based features instead of collecting independent clinical text or patient records that provide domain-informed complementary features to capture clinically relevant lesion characteristics. In the future, we will focus on real-world clinical symptom textual data to further improve model generalization and the framework’s multimodal capability. Overall, the framework not only enhances diagnostic accuracy but also creates a robust, adaptable system that can support dermatologists in delivering timely and precise care for a wide range of skin conditions.

## Data Availability

The original contributions presented in the study are included in the article/**Supplementary Material**. Further inquiries can be directed to the corresponding author.

## References

[1] Liu R Chen Z Yao G Zhang P . Exploring the challenge and value of deep learning in automated skin disease diagnosis. BioMed Signal Process Control. (2026) 117:109589. doi: 10.1016/j.bspc.2026.109589 38826717

[2] Lilhore UK Sharma YK Simaiya S Alroobaea R Baqasah AM Alsafyani M . SkinEHDLF a hybrid deep learning approach for accurate skin cancer classification in complex systems. Sci Rep. (2025) 15:14913. doi: 10.1038/s41598-025-98205-7 40295588 PMC12037884

[3] Sethy PK Behera SK Kannan N . Categorization of common pigmented skin lesions (CPSL) using multi-deep features and support vector machine. J Digital Imaging. (2022) 35:1207–16. doi: 10.21203/rs.3.rs-136988/v1 35524077 PMC9582098

[4] Kassem MA Hosny KM Damaševičius R Eltoukhy MM . Machine learning and deep learning methods for skin lesion classification and diagnosis: a systematic review. Diagnostics. (2021) 11:1390. doi: 10.3390/diagnostics11081390 34441324 PMC8391467

[5] Gairola AK Kumar V Sahoo AK Diwakar M Singh P Garg D . Multi-feature fusion deep network for skin disease diagnosis. Multimedia Tools Appl. (2025) 84:419–44. doi: 10.1007/s11042-024-18958-7 30311153

[6] Vasanthakumari P Romano RA Rosa RG Salvio AG Yakovlev V Kurachi C . Discrimination of cancerous from benign pigmented skin lesions based on multispectral autofluorescence lifetime imaging dermoscopy and machine learning. J BioMed Opt. (2022) 27:066002. doi: 10.1117/1.jbo.27.6.066002 35701871 PMC9196925

[7] Gupta P Nirmal J Mehendale N . A survey on computer vision approaches for automated classification of skin diseases. Multimedia Tools Appl. (2025) 84:8673–705. doi: 10.1007/s11042-024-19301-w 30311153

[8] Esteva A Kuprel B Novoa RA Ko J Swetter SM Blau HM . Dermatologist-level classification of skin cancer with deep neural networks. Nature. (2017) 542:115–8. doi: 10.1038/nature21056 28117445 PMC8382232

[9] Zareen SS Hossain MS Wang J Kang Y . Recent innovations in machine learning for skin cancer lesion analysis and classification: a comprehensive analysis of computer‐aided diagnosis. Precis Med Sci. (2025) 14:15–40. doi: 10.1002/prm2.12156 41531421

[10] Nimesh V Weerasinghe R . Differential diagnosis of ringworm and eczema using image processing and deep learning. 2021 21st Int Conf Adv ICT Emerging Regions (ICter). (2021), 147–52. doi: 10.1109/ICter53630.2021.9774803 25079929

[11] Joseph N Kumar GS Nelliyanil M . Skin diseases and conditions among students of a medical college in southern India. Indian Dermatol Online J. (2014) 5:19–24. doi: 10.4103/2229-5178.126023 24616849 PMC3937480

[12] Liu Z Pan H Gong C Fan Z Wen Y Jiang T . Multi-class skin lesion segmentation for cutaneous T-cell lymphomas on high-resolution clinical images. MICCAI. (2020), 351–61. doi: 10.1007/978-3-030-59725-2_34 28118817

[13] Zhang J Petitjean C Ainouz S . Kappa loss for skin lesion segmentation in fully convolutional network. IEEE ISBI. (2020), 2001–4. doi: 10.1109/ISBI45749.2020.9098404 25079929

[14] Ali SN Ahmed MT Paul J Jahan T Sani SMS Noor N . Monkeypox skin lesion detection using deep learning models: a feasibility study. In: Arxiv Preprint Arxiv:2207.03342. Ithaca, New York, USA: Cornell University (2022). doi: 10.48550/arXiv.2207.03342

[15] Murthy AN Krishnamaneni R Rao TP Vidyasagar V Ambhika C Padmaja IN . Deep long and short term memory with tunicate swarm algorithm for skin disease detection and classification. J Electrical Syst. (2024) 20:613–24. doi: 10.52783/jes.3372

[16] Singh J Sandhu JK Kumar Y . An analysis of detection and diagnosis of different classes of skin diseases using artificial intelligence-based learning approaches with hyper parameters. Arch Comput Methods Eng. (2024) 31:1051–78. doi: 10.1007/s11831-023-10005-2 30311153

[17] Jusman Y Firdiantika IM Dharmawan DA Purwanto K . Performance of multi layer perceptron and deep neural networks in skin cancer classification. In: IEEE Lifetech. Piscataway, New Jersey: IEEE (2021). p. 534–8. doi: 10.1109/LifeTech52111.2021.9391876

[18] Likhitha S Baskar R . Skin cancer classification using CNN in comparison with support vector machine for better accuracy. In: Smart. Piscataway, New Jersey: IEEE (2022). p. 1298–302. doi: 10.1109/SMART55829.2022.10047280

[19] Barbadekar A Ashtekar V Chaudhari A . Skin cancer classification and detection using VGG-19 and DesNet. In: Iccins. Piscataway, New Jersey: IEEE (2023). p. 1–6. doi: 10.1109/ICCINS58907.2023.10449996

[20] Ahmed T Mou FS Hossain A . SCCNet: an improved multi-class skin cancer classification network using deep learning. In: Icaeee. Piscataway, New Jersey: IEEE (2024). p. 1–5. doi: 10.1109/ICAEEE62219.2024.1056167

[21] Dosovitskiy A . An image is worth 16x16 words: transformers for image recognition at scale. In: Arxiv Preprint Arxiv:2010.11929. Ithaca, New York: Cornell University (2020). doi: 10.48550/arXiv.2010.11929

[22] Yang G Luo S Greer P . A novel vision transformer model for skin cancer classification. Neural Process Lett. (2023) 55:9335–51. doi: 10.1007/s11063-023-11204-5 30311153

[23] Xu R Wang C Zhang J Xu S Meng W Zhang X . SkinFormer: learning statistical texture representation with transformer for skin lesion segmentation. IEEE J BioMed Health Inform. (2024) 28:6008–18. doi: 10.1109/JBHI.2024.3417247 38913520

[24] Wang S Yin Y Wang D Wang Y Jin Y . Interpretability-based multimodal convolutional neural networks for skin lesion diagnosis. IEEE Trans Cybern. (2021) 52:12623–37. doi: 10.1109/TCYB.2021.3069920 34546933

[25] Li Y Yang X Chen Y Chen S Zeng B Cai H . Adaptive multi-modal data fusion method for dermatosis diagnosis. IEEE BIBM. (2023), 2904–11. doi: 10.1109/BIBM58861.2023.10385866 25079929

[26] He X Wang Y Zhao S Chen X . Co-attention fusion network for multimodal skin cancer diagnosis. Pattern Recognit. (2023) 133:108990. doi: 10.1016/j.patcog.2022.108990 38826717

[27] Zhou L Luo Y . Deep features fusion with mutual attention transformer for skin lesion diagnosis. IEEE ICIP. (2021), 3797–801. doi: 10.1109/ICIP42928.2021.9506211 25079929

[28] Tschandl P Rosendahl C Kittler H . The HAM10000 dataset, a large collection of multi-source dermatoscopic images of common pigmented skin lesions. Sci Data. (2018) 5:180161. doi: 10.1038/sdata.2018.161 30106392 PMC6091241

[29] Codella N Rotemberg V Tschandl P Celebi ME Dusza S Gutman D . Skin lesion analysis toward melanoma detection 2018: a challenge hosted by the international skin imaging collaboration (ISIC). In: Arxiv Preprint Arxiv:1902.03368. Ithaca, New York: Cornell University (2019). doi: 10.48550/arXiv.1902.03368

[30] ISIC . Skin lesion analysis towards melanoma detection (2018). Available online at: https://challenge.isic-archive.com/data/ (Accessed August 13, 2026).

[31] ISIC . Archive. Available online at: https://www.isic-archive.com (Accessed August 13, 2026).

[32] Friedman RJ Rigel DS Kopf AW . Early detection of Malignant melanoma: the role of physician examination and self-examination of the skin. CA Cancer J Clin. (1985) 35:130–51. doi: 10.3322/canjclin.35.3.130 3921200

[33] Amiri SA Nasrolahzadeh M Mohammadpoory Z Kordkheili AHZ . Skin lesion classification via ensemble method on deep learning. Multimedia Tools Appl. (2025) 84:19379–97. doi: 10.1007/s11042-024-19837-x 30311153

[34] Khan AR Mujahid M Alamri FS Saba T Ayesha N . Early‐stage melanoma cancer diagnosis framework for imbalanced data from dermoscopic images. Microsc Res Tech. (2025) 88:797–809. doi: 10.1002/jemt.24736 39573895

[35] Ali MD Mazhar T Shahzad T Rehman WU Shahid M Hamam H . An advanced deep learning framework for skin cancer classification. Rev Socionetwork Strategies. (2025) 19:111–30. doi: 10.1007/s12626-025-00181-x 30311153

[36] Kingni ST Baawain S . Comparative evaluation of convolutional neural network architectures for automated skin cancer classification: a study on the ISIC 2018 dataset. Artif Intell Appl Sci. (2026) 2:8–14. doi: 10.69882/adba.ai.2026012

[37] Liu Y Chen L Liu J Zhu M Zhong Z Yu B . ViSurf: visual supervised-and-reinforcement fine-tuning for large vision-and-language models. In: Arxiv Preprint Arxiv:2510.10606. Ithaca, New York: Cornell University (2025). doi: 10.48550/arXiv.2510.10606

