# Supplementary material for: Visual and lesion descriptor fusion using cross-attention for skin disease diagnosis
Source: Front Oncol. 2026 Aug 21;16:1885137. doi: 10.3389/fonc.2026.1885137 (PMC13541541; doi:10.3389/fonc.2026.1885137)
Supplement: Supplementary file 1 [file Table1.docx]

Supplementary Material

## 1.1 Supplementary Data

The supplementary material associated with this article includes the generated structured lesion descriptor dataset used to support multimodal skin disease classification. The supplementary data were derived from the publicly available ISIC 2018 dermoscopic image dataset through descriptor generation based on lesion characteristics including size, shape, color, and texture attributes.

The supplementary dataset is publicly available at:

**Figshare Repository:**
https://doi.org/10.6084/m9.figshare.32314554
